# Experimental application of Business Process Management technology to manage clinical pathways: a pediatric kidney transplantation follow up case

**DOI:** 10.1186/s12911-017-0546-x

**Published:** 2017-11-03

**Authors:** Martina Andellini, Sandra Fernandez Riesgo, Federica Morolli, Matteo Ritrovato, Piero Cosoli, Silverio Petruzzellis, Nicola Rosso

**Affiliations:** 1 Health Technology Assessment Unit, Bambino Gesù Children’s Hospital, Viale Ferdinando Baldelli, 41, 00146 Rome, Italy; 2 IT Department, Bambino Gesù Children’s Hospital, Rome, Italy; 3 Surgery Department, Bambino Gesù Children’s Hospital, Rome, Italy; 4 Operations Director, Openwork srl, Bari, Italy; 5 Product Manager, Openwork srl, Bari, Italy; 6 IT Department, Bambino Gesù Children’s Hospital, Rome, Italy

**Keywords:** Clinical pathway, Kidney transplantation, Pediatrics, Case management, Business process management

## Abstract

**Background:**

To test the application of Business Process Management technology to manage clinical pathways, using a pediatric kidney transplantation as case study, and to identify the benefits obtained from using this technology.

**Methods:**

Using a Business Process Management platform, we implemented a specific application to manage the clinical pathway of pediatric patients, and monitored the activities of the coordinator in charge of the case management during a 6-month period (from June 2015 to November 2015) using two methodologies: the traditional procedure and the one under study.

**Results:**

The application helped physicians and nurses to optimize the amount of time and resources devoted to management purposes. In particular, time reduction was close to 60%. In addition, the reduction of data duplication, the integrated event management and the efficient data collection improved the quality of the service.

**Conclusions:**

The use of Business Process Management technology, usually related to well-defined processes with high management costs, is an established procedure in multiple environments; its use in healthcare, however, is innovative. The use of already accepted clinical pathways is known to improve outcomes. The combination of these two techniques, well established in their respective areas of application, could represent a revolution in clinical pathway management. The study has demonstrated that the use of this technology in a clinical environment, using a proper architecture and identifying a well-defined process, leads to real benefits in terms of resources optimization and quality improvement.

## Background

During recent years, two of the most important targets in healthcare have been the optimization of resources and the improvement of efficiency and quality.

These objectives have become an important goal in view of the limited ability of health organizations to adopt Information Technology (IT) due to the complexity of these organizations and their fragmented internal structures. In other words, healthcare institutions have been managed individually using “ad hoc” solutions. The main critical elements in healthcare management are the variability due to different availability of healthcare services, the scarce use of medical evidence and the phenomena of professional uncertainty. This has led to a slow evolution in healthcare management and optimization because the new methodologies, technologies and practices have not been implemented in healthcare environments at the same rate as in other sectors [1]. This aspect is critical because the main objective of health care services is to reduce costs without reducing the quality in patient care. To reach the best cost-quality ratio, healthcare management has to reach the same level as other services in terms of efficiency and, to do this, we will need to follow the same path as the others, using the new technologies and methodologies available.

One of the methodologies developed and established internationally since the 1980s is the implementation of clinical pathways to guide evidence-based healthcare [2]. Clinical pathway are clinical management tools used to manage the quality in health care concerning the standardization of care processes. Clinical pathways are an evidence-based response to specific health care problems and provide physicians and nurses with a pre-established care plan devised according to international standards developed by specialists in the field. The application of these standards helps professionals to offer patients the best possible care plan, without needing to devise an individual care plan for each patient, thus avoiding human error and saving time for both physicians and patients [3].

The management of these care plans is often a complex task as the pathway integrates several guidelines, with different tests that have to be performed at specific time points. Clinical pathway timeline defines the expected plan of treatments for a group of patients with a particular diagnosis or undergoing a particular clinical procedure. It outlines time-specified plan of treatment recommending tests and therapies based upon a combination of clinical practice consensus and evidence from the scientific literature, thus leading to an improvement in the quality of clinical outcomes [4]. When the pathway is applied to a large set of patients, management becomes a time-consuming activity. It has been shown that their implementation reduces the variability in clinical practice and improve clinical outcomes [4].

All the tests planned for the pathway must be coordinated according to the resources available and to the pathway schedule, including the specific variations according to the clinical situation of the patient.

Adverse events are another parameter to be included in the case management. When a patient experiences an adverse event, specific guidelines or protocols are followed.

With the above premises, we hypothesized that using Business Process Management (BPM) techniques would improve the coordination task.

Business Process Management is a discipline that has evolved since the 1990s and has been adopted in many environments to manage and optimize end-to-end processes. In healthcare, these techniques have only been used during the last two decades [5] since they provide support at the business or organization level and are usually applied to well-defined processes. In the clinical environment, the integration of this tool is innovative. A literature search, limited to PubMed, found very few articles (4) using the terms “pathways” and “business process management” and none of them described a practical implementation.

## Methods

The aims of this work were to define a clinical pathway as a process, using a pediatric kidney transplantation as a case study, and to identify the benefits obtained from using this technology by monitoring particular characteristics and many parameters. We sought to have a wider view of the complex clinical process and to manage each patient case efficiently, integrating events and variations for each individual situation. We believed that achieving this aim would lead to an increase in the quality of the work carried out by healthcare workers and a decrease in workload.

We have not limited our project to abstractions; it also includes concrete and practical aspects. We have developed a web-based application to integrate the BPM approach in the management of clinical pathways.

### The context

The standard BPMN has been already used at Bambino Gesù Children’s Hospital (Rome, Italy) for a previous project, whose objective was the optimization and planning of operating theatre activities [6]. Bambino Gesù Children’s Hospital is the largest Italian pediatric hospital managing high complexity cases of kidney transplantation.. In the hospital, transplants are performed in children with more than 8 kg body weight. All aspects of childcare are taken into account through an integrated and multidisciplinary approach, including psychological development [7].

During each patient admission, either in the day hospital or in the ward, a member of the transplant team has to:

- search the patient data in the hospital repository (personal data, medical reports, laboratory analysis results and follow up plan)
- select only clinical data relevant for decision-making - creating for instance special filters on the results
- save the data in a shared document in specific folders of the file system, following an internal coding standard for classifying information

This means that for each patient at each visit, the transplant coordinator has to check which exams have already been performed and what is still to be done, and create a specific program for each patient. A patient may miss an appointment for several reasons and, in this case, another appointment has to be planned and the management of patient lists needed to make rearrangements. Moreover, results are currently stored in a database which does not allow direct downloading from the hospital system.

To monitor the entire pathway of a single patient, the clinical team needs to navigate the clinical repository to access all the relevant data and copy them into a shared folder, including the specific documents for kidney transplantation.

Coping with the variability of the process is therefore a matter of considerable manual file manipulation, with a difficult-to-manage and error-prone information-sharing practice. Currently we have a list of tests (laboratory, instrumental, specialist visits) that must be performed every year at a specific time point according to international guidelines [8–14]. Feeding the database is thus a time-consuming task for the transplant coordinator.

Having a computerized system with a specifically designed database, which interacts with the hospital clinical repository, should allow a prompt retrieval of patient data. This will be of great help in guiding clinical decisions and in carrying out research projects. In addition, scheduling patients’ appointments could be automatic, reducing human mistakes and leading to time saving.

As a result, this process was selected for the experimental application of BPM technology to prove the feasibility of its implementation and identify the benefits deriving from its use.

Several techniques and practices based on new technology and innovative patterns and approaches have been used during recent years in an attempt to attain the optimization goal. One of these is the above-mentioned Business Process Management (BPM).

BPM is a discipline designed to improve processes and help organizations to manage their activities efficiently. It involves any combination of modeling, automation, execution, control, measurement and optimization of flows, as stated in the definition given by Nathaniel Palmer [15].

BPM is intended for analysis and design, and uses a specific notation recognized as an international standard: Business Process Modeling Notation (BPMN 2.0). We used the latest version, BPMN 2.0 for the analysis and design phases of the project [16].

### Approach

Once a pathway had been identified, interviews and meetings were held to share knowledge of the case with the IT Team whose first responsibility was the analysis of the case and the process definition. To complete the global analysis of the case, more interviews were held to elicit the requirements of the physicians and nurses of the coordination center. Once all the information necessary for the application had been obtained, the team was able to produce a document defining the process that would be managed automatically by the application and meet the requirements of the final users.

The next task was to translate all the information and requirements into a design document, directed towards the implementation of the application using the Jamio platform; for the process definition, we used the BPMN 2.0 standard [16].

The design document was used as input for the implementation of the application. The Jamio platform offers a BPM editor, called *Composer*, which uses the BPMN 2.0 to create processes. This tool allows the user to reproduce the whole domain: the different kinds of users, the data types, the relationships, the rules, the layout, etc.

After the first version of the implementation, iterations were discussed with the final users with the aim to improve the satisfaction level and to offer the users the opportunity to become familiar with the tool.

When the final version was delivered, the team organized a presentation with the technological partners and medical teams to share the results of all the work done.

Several iterations suggested by the users have been implemented since then.

### Technology

To develop the application, we have used the Jamio Platform (Openwork srl), which integrates the BPMN 2.0 in its modeling tool for design and implementation [16].

The system was selected to meet key requirements of both the user panel and the IT department:

- the possibility for clinicians to design the standard basic protocol without the involvement of IT people
- the possibility to cope with unexpected events occurring during care path activities
- the possibility to access and integrate clinical repository data during follow-up activities
- the possibility to involve different stakeholders, belonging to different organizations or institutions
- the possibility to analyze clinical data collected during the entire course of the care path
- the possibility to monitor and analyze the cost of activities during follow-up

The system was implemented using a BPM-based platform with a service-oriented architecture (SOA) to facilitate access to the clinical repository using web services. Moreover, the platform provided integrated human task management capabilities and was able to orchestrate services with an event-based approach. The platform’s model-driven approach allowed the detailed specification of protocols by clinicians and the integrated recording of the scheduled activities by the appointed stakeholders. This enabled the system to embrace and manage both planned actions and unexpected events diverging from the protocols predefined by clinicians.

The whole set of activities, both those planned in advance and those resulting from an unexpected event that could influence follow-up, can be assigned to the appropriate actors according to the activity to be performed, thanks to the capacity of the integrated organization management services to map virtually the organization of the departments involved in the case.

The BPM-based platform supports the follow-up process design completely, involving both the clinicians and the IT department, allowing them to manage their respective domains with the appropriate instruments.

### Process analysis

**Fig. 1:**
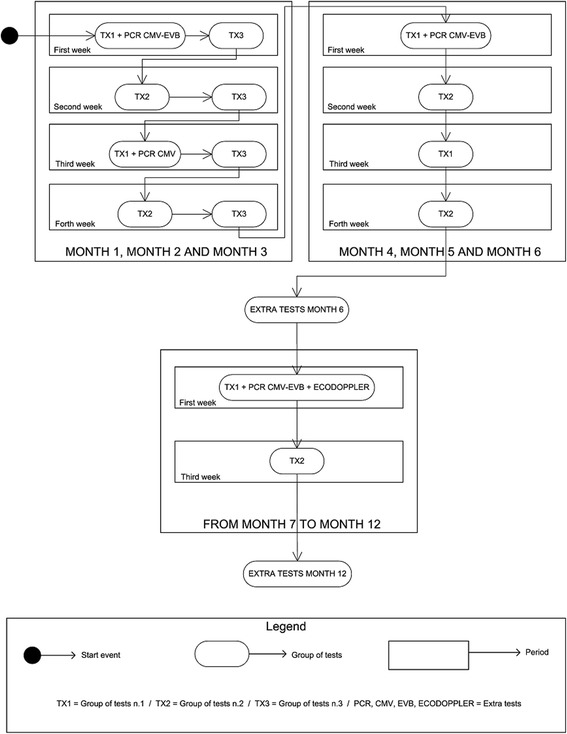
shows the first part of the clinical protocol foreseen for the first year after transplantation. Each group of tests to be performed is planned for a specific period, their frequency varying throughout the process. The filled circle represents the starting point for the process. The rectangles with rounded corners represent tasks or activities, and in this case represent the group of tests to be performed. The rectangles in the background layer represent the periods when the tasks are performed

**Fig. 2:**
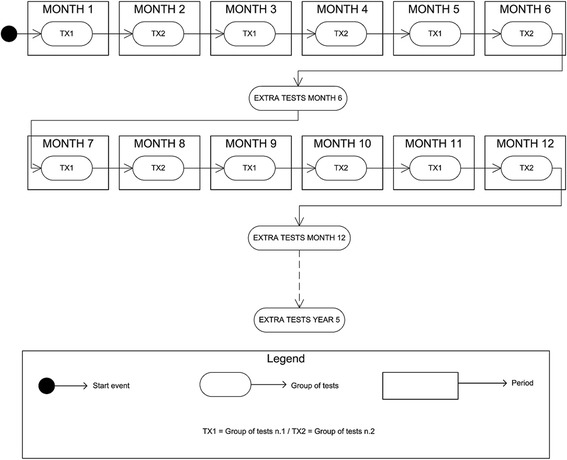
shows the cyclical process to be followed after completing the first year protocol. The combination of the two figures shows the entire clinical pathway applied to the patients, which is the final process to be managed by the BPM application. These two activity diagrams were the main output of the process definition phase.The filled circle represents the starting point for the process. The rectangles with rounded corners represent tasks or activities, and in this case represent the group of tests to be performed. The rectangles in the background layer represent the periods when the tasks are performed

This study is an example of Case Management implementation, so the first task was to assemble all available knowledge concerning the case. The starting point for this purpose was the standard monitoring protocol applied to each patient. Based on the stakeholders’ knowledge and the documents provided, we have created a process to represent the clinical pathway. Figure 1 shows the first part of the clinical protocol foreseen for the first year after transplantation. Each group of tests to be performed is planned for a specific period, their frequency varying throughout the process. Figure 2 shows the cyclical process to be followed after completing the first-year protocol. The combination of the two figures shows the entire clinical pathway applied to the patients, which is the final process to be managed by the BPM application. These two activity diagrams were the main output of the process definition phase.

Although these diagrams show a well-defined monitoring plan, in real life most of the protocols are modified according to the clinical situation of each patient. This means that the main requirement is flexibility and possibility to customize on a per-patient basis each process at run time after the automatic creation of the “template care plan”. The case shown is quite complex from this point of view due to the fact that an event may occur at any moment and the consequences are only known when the clinical team makes a decision on how to manage them.

The necessity of having a flexible process was the main reason for adopting the innovative architecture. Usually, when using BPM, the process is well defined and static, but this is not the case. For these reasons, we defined a proper architecture and identified a well-defined process to improve the flexibility that fit our need. The process that manages the pathway, instead of being a complex single process, is divided into three main managing processes.

- The first one receives the medical visit templates introduced by the administrator and generates the default pathway for the patient.
- The second process runs daily and creates the human tasks and visit templates whose dates are included in the planning period.
- The third one manages the actions to perform when the user creates a new patient event in the platform solution.

### Evaluation methods

For this initial evaluation, time reduction was selected as the main parameter for evaluating the application introduction.

In order to evaluate the time reduction, the experiment proposed was to manage with the new application a set of 22 patients, selected from among all the patients. By monitoring the daily activity of the coordinator for the two groups, i.e., the 22 patients managed with the application and the rest (248) with the traditional method, we were able to compare the time taken to manage the integrated clinical pathway for each patient in the two groups.

During 6 months while the experimental phase was active, we monitored with a stopwatch the time required to complete every activity for each patient under both types of management that we named “Current Management” and “BPM Management”.

To obtain the average time-reduction percent (Eq. 1) we used the average time spent per patient with the BPM Management and the average time spent per patient with the current management. These two values represent the average total time spent per patient to manage the clinical pathway, including the retrieval of laboratory results, planning next admissions and communicating the planning to the stakeholders within the department. The average time-reduction percent estimates the average time saved by the users in the daily clinical pathways management tasks.

1 $$ {TRp}_{AV}=\left(1-\frac{T_{Av}^{BPMM}}{T_{Av}^{CM}}\right)\times 100 $$

with TAvBPMM being the average time per patient with the BPM management and TAvCM being the average time per patient with the current management.

## Results

### Time reduction evaluation

As mentioned above, in this study we have monitored the activities of the main stakeholder, the transplant coordinator, for 6 months. During this period, using a stopwatch the coordinator recorded the time spent doing management tasks for 30 effective days.

In order to compare results between the two managing methods, 22 patients were selected to have their clinical pathway managed with the new application. The remaining patients were managed with the current methods.

**Fig. 3:**
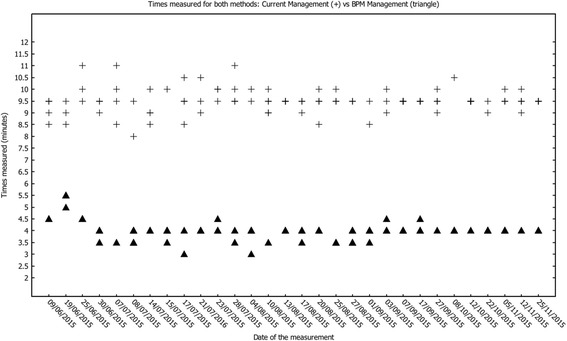
shows the statistical results obtained during the monitoring period to estimate the average time-reduction percentage for both methods: current management and BPM method. The total number of values recorded was 173. Eighty time-measurements were recorded for using the BPM application, while 93 were recorded for traditional management. Each point represents a time-measurement for the management of the follow up of a single patient. Each cross represents one measurement of the total time spent for the management of a single patient’s follow-up performed with the traditional method. Each filled triangle represents one measurement of the total time spent for the management of a single patient’s follow-up performed with the BPM method

Figure 3 shows the statistical results obtained during the monitoring period and which were used to calculate the two average values required in Eq. 1 to determine the average time-reduction percentage. The total number of values recorded was 173. Eighty (80) time-measurements were recorded for use with the BPM application, while 93 were recorded for traditional management. Each point in Fig. 3 represents a time-measurement for the management of the follow up of a single patient. Some points in the figure may overlap with others.

**Fig. 4:**
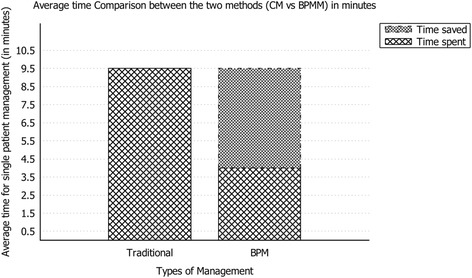
shows the average time-reduction comparing the different times for the two methods. The percentage of time saved by using the new method is 57.9% (p<0.001), the average time for the BPM method is 4 minutes, while the coordinator needs 9.5 minutes with the traditional method (Lab time reduction is equal to 37.5%, p<0.001; Com time reduction is equal to 100%, as the task is no longer performed; Plan time reduction is equal to 70.0%, p<0.001)

Figure 4 shows the average time-reduction comparing the different times for the two methods. The percentage of time saved by using the new method is 57.9% (*p* < 0.001), the average time for the BPM method is 4 min, while the coordinator needs 9.5 min with the traditional method (Lab time reduction is equal to 37.5%, *p* < 0.001; Com time reduction is equal to 100%, as the task is no longer performed; Plan time reduction is equal to 70.0%, *p* < 0.001).

Focusing on the detailed tasks involved in the management activities, Table 1 provides further data on the time spent for each particular task. Three different tasks need to be performed to manage the patient’s entire follow-up: retrieving laboratory results for the latest tests (Lab), planning the next tests to be performed according to the pathway (Plan), and communication of the planning to the department staff in charge of the tests (Com).

**Table 1:** Arithmetic mean, Standard deviation and Coefficient of variation of each task measurement and totals

	Traditional Management				BPM Management			
	Lab	Com	Plan	Total	Lab	Com	Plan	Total
Arithmetic mean (in minutes)	4	0.5	5	9,5	2.5	0	1.5	4
Stardard Deviation	0.21	0.00	0.26	0.35	0.19	0.00	0.22	0.35
Coefficient of Variation	0.04	0.00	0.07	0.12	0.04	0.00	0.05	0.12

In particular, Table 1 shows that communication time with the BPM management is zero; in fact, with the application the communication task is automatic. In other words, the operator spends no time on this activity.

Finally, results exhibit important reduction of the range of time spent per patient managed with BPM [3–5,5 min] compared to the range of time spent per patient managed with current method [8–12 min].

### Other benefits obtained

The new management method has brought benefits other than the time reduction. However, in this study, other parameters have not been quantified.

There are certain quality improvements with the use of BPM technology compared to the current method, for example the efficient automatic e-mail planning for the nurses who perform the medical exams for patients. It can reduce human errors, as the planning contains all the information necessary for preparing resources. The automatic management of admission appointments and the associated protocols is another example. It reduces human errors such as forgetting appointments or saving wrong data. This means that the quality of data and reliability of the pathway scheduling have been seriously improved. Other important improvements for daily activities are that the BPM technology offer the possibility to gather every kind of data filtered for monitoring and to obtain simple reports with the exam results in different document formats.

Data accuracy has also increased which avoids data duplication by using the automatic import from the internal repository when required.

## Discussion

The center for Transplant Coordination was created to focus on the pathway management of almost 300 patients. This project was presented as an initiative to reduce the staff workload. The coordinator will have just one point of access for all the necessary filtered information and will use the automatically set default schedule. This is an example of how Information Communication Technologies (ICT) can be used for optimization purposes in this environment.

ICT are widely exploited in the healthcare domain, with a main focus on Electronic Health Records (EHR) or Telemedicine. However, both these applications consist mainly of showing patient records or gathering information that is “pure provision of data”. To organize the sequence of tasks for a patient-specific treatment pathway in a proper and rigorous manner is a rather different project. Such projects, based on BPM-based Case Management technology, are an important step towards improving healthcare support platforms, where decision-making and knowledge-management are supported in a sustainable framework that allows the coordination of multi-disciplinary care-processes.

The use of BPM technology, combined with the adoption of standard medical protocols, is quite an innovation in hospitals all over the world. This structured approach to clinical work can lead to relevant cost reductions and better outcomes, but most of all can provide leverage for clinical process control as well as the possibility to study the gaps between standard protocols and specific clinical complexities.

In this study, we evaluated the implementation of clinical pathway for the management of pediatric kidney transplantation, and compared the outcomes of patients managed with BPM technology and with standard care guidelines. To evaluate the time spent to manage the clinical pathway of the patient’s entire follow-up, three different tasks were performed including the retrieval of laboratory results, planning next admissions and communicating the planning to the stakeholders within the department.

This study confirmed our hypothesis that the usage of BPM in the management of kidney transplantation leads to real benefits in terms of resources optimization and quality improvement. The time to manage kidney transplantation with BPM technology is statistically significant less than the current method.

The percentage of time saved by using the new method is 57.9% (*p* < 0.001). Laboratory time reduction is equal to 37.5%, the time reduction in planning next admissions is equal to 100% and the reduction time communicating the planning to the stakeholders within the department is equal to 70.0%.

This study also highlights the quality improvements due to the use of BPM technology. The reduction of human errors is one of the main improvement related to automatic management of admission appointments and the associated protocols, and to automatic e-mail planning for the nurses. This means that the quality of data and reliability of the pathway planning have been improved.

Finally, the fact of having a tool which gathers every kind of data filtered for monitoring is a relevant quality improvement for the department, since the staff can focus on the patient and not on data retrieval.

The use of already accepted clinical pathways is known to improve outcomes [3]. BPM technology can benefit from approaches and techniques at several stages of the business process life cycle in order to increase dynamism, flexibility and competitiveness. The combination of BPM-based platform with a service-oriented architecture could represent a revolution in clinical pathway management.

### Limitations and future work

As an exploratory work, our study has a number of limitations. We selected only one case for BPM management, although we did create a prototype application, which could be easily extended, without redeployment, to manage other clinical pathways within the hospital.

If extra resources were available, a more profound analysis focusing on different parameters could be proposed to investigate the improvement of aspects other than time reduction. The development of models to analyze costs and variations would be an interesting undertaking.

At the Bambino Gesù Children’s Hospital, we have begun further analyses to identify possible new clinical pathways where these techniques could be applied.

## Conclusions

This study investigated the feasibility of applying BPM techniques for the management of clinical pathways. The results showed that the different stakeholders obtained different benefits in terms of quality and efficiency. In particular, the implementation of this application led to an important reduction in the time needed by staff to carry out the activities of the Transplant Coordination Centre as well as a reduction of the workload. The preliminary results of this study may be used for the further development of the use of these techniques in the clinical environment.

## References

[1] England I, Stewart D, Walker S (2000). Information technology adoption in health care: when organisations and technology collide. Aust Health Rev.

[2] Kinsman L, Rotter T, James E (2010). What is a clinical pathway? Development of a definition to inform the debate. BMC Med.

[3] anella M, Marchisio S, Di Stanislao F. Reducing Clinical variations with clinical pathways: do pathways work? Int J Qual Health Care. 2003;15(6):509-521. 10.1093/intqhc/mzg057 14660534

[4] Panella M (2003). Reducing clinical variations with clinical pathways. Do pathway work?. Int J Qual Health Care.

[5] González Sánchez MJ, Framiñán Torres JM, Parra Calderón CL (2008). Application of business process management to drive the deployment of a speech recognition system in a healthcare organization. Stud Health Technol Inform.

[6] Barbagallo S, Corradi L, de Ville de Goyet J (2015). Optimization and planning of operating theatre activities: an original definition of pathways and process modeling. BMC Med Inform DecisMak.

[7] Tozzi AE, Mazzotti E, Di Ciommo VM (2012). Quality of life in a cohort of patients diagnosed with renal failure in childhood and who received renal transplant. Pediatr Transplant.

[8] Cohen D, Galbraith C (2001). General health management and long-term care of the renal transplant recipient. Am J Kidney Dis.

[9] Adams PL (2006). Long-term patient survival: strategies to improve overall health. Am J Kidney Dis.

[10] Howard AD (2006). Long-term posttransplantation care: the expanding role of community nephrologists. Am J Kidney Dis.

[11] Josephson MA (2011). Monitoring and managing graft health in the kidney transplant recipient. Clin J Am Soc Nephrol.

[12] Hariharan S (2006). Recommendations for outpatient monitoring of kidney transplant recipients. Am J Kidney Dis.

[13] Kasiske BL, Vazquez MA, Harmon WE (2000). Recommendations for the outpatient surveillance of renal transplant recipients. American Society of Transplantation. J Am Soc Nephrol.

[14] Howard AD (2001). Long-term management of the renal transplant recipient: optimizing the relationship between the transplant center and the community nephrologist. Am J Kidney Dis.

[15] Nathaniel P. What is BPM? BPM.com. http://bpm.com/what-is-bpm (accessed 4 Feb 2016).

[16] Documents Associated with Business Process Model and Notation (BPMN) Version 2.0. http://www.omg.org/spec/BPMN/2.0/PDF/ (accessed 4 Feb 2016).

